# Neoadjuvant chemotherapy for primary sarcoma of the breast: a case report

**DOI:** 10.1186/s13256-019-2197-2

**Published:** 2019-09-06

**Authors:** Chieko Miyazaki, Mikio Shiozawa, Rintaro Koike, Kasumi Ogihara, Yumiko Sasaki, Satomi Shiba, Saki Nishida, Masako Sakuragi, Hirofumi Mizunuma, Takashi Fujita, Noriyoshi Fukushima, Alan K. Lefor, Joji Kitayama, Naohiro Sata

**Affiliations:** 1 The Department of Breast Surgery, Jichi Medical University, Yakushiji 3311-1, Shimotuke, Tochigi, 329-0498 Japan; 2 The Department of Surgery, Medical Center Shimotsuga, Ohiramachi Kawazure 420-1, Tochigi, Tochigi 329-4498 Japan; 3 The Department of Pathology, Jichi Medical University, Yakushiji 3311-1, Shimotuke, Tochigi, 329-0498 Japan; 4 The Department of Surgery, Jichi Medical University, Yakushiji 3311-1, Shimotuke, Tochigi, 329-0498 Japan

**Keywords:** Breast, Leiomyosarcoma, Neoadjuvant chemotherapy

## Abstract

**Background:**

Primary sarcoma of the breast is rare. Surgery has been the only curative treatment available. Recently, neoadjuvant chemotherapy including anthracycline/ifosfamide has been reported effective for patients with high-risk sarcomas in a prospective trial.

**Case presentation:**

A 52-year-old Japanese woman presented with a mass in her left breast. The 10 cm tumor was fixed to her chest wall on examination. A skin biopsy was performed which showed leiomyosarcoma. Neoadjuvant chemotherapy was given and the tumor became mobile. A mastectomy and axillary dissection were performed with surgically negative margins. After neoadjuvant chemotherapy, the amount of necrosis was profoundly influenced by chemotherapy, and the histological effect of neoadjuvant chemotherapy was assessed in reference to pre-neoadjuvant chemotherapy magnetic resonance imaging.

**Conclusion:**

In contrast to many other cancers, the evaluation of various treatments and of the histological effect of neoadjuvant chemotherapy for sarcoma has been difficult due to the rarity of these tumors. We report the case of a patient with a breast sarcoma, treated with neoadjuvant chemotherapy and discuss the appropriate pathological evaluation and therapeutic management.

## Introduction

Breast sarcoma is a rare entity; it is a heterogeneous group of uncommon neoplasms arising from mesenchymal tissues of the breast. Breast sarcomas accounted for 0.0006% of all breast malignancies [1]. Over the past decade, the number of cases of breast sarcoma after breast irradiation for previous breast carcinoma has increased [2–4].

Surgery with an adequate resection margin is the only potentially curative therapy for patients with sarcomas. However, a recent randomized trial provided evidence to support the use of neoadjuvant chemotherapy for patients with high-risk soft tissue sarcomas of the extremities and trunk by European sarcoma groups [5, 6]. We describe here a case of breast sarcoma treated with neoadjuvant chemotherapy and successful resection. The case is discussed with a review of appropriate pathological evaluation and therapeutic management.

## Case presentation

**Fig. 1:**
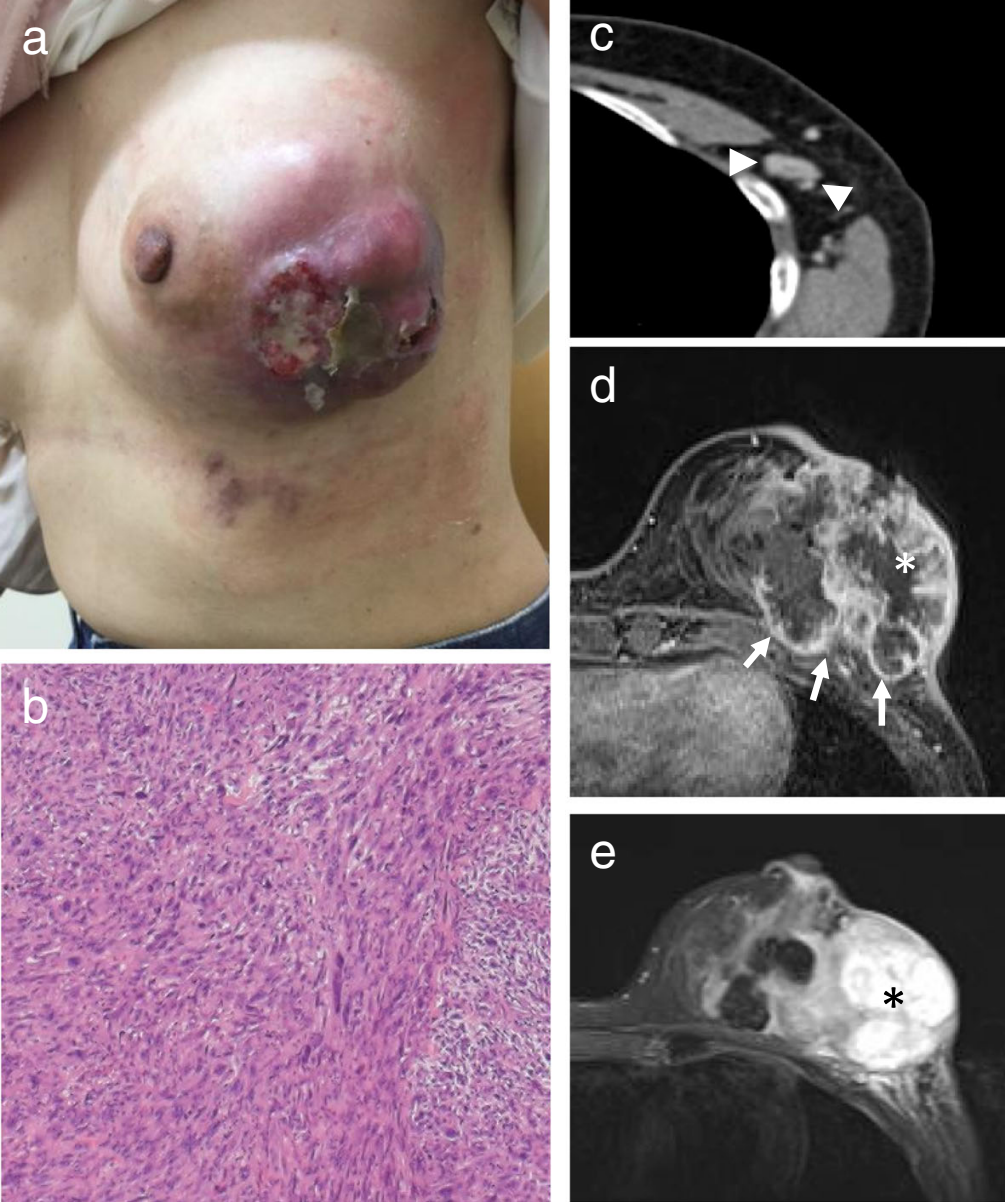
**a** The bulky mass with ulceration prior to neoadjuvant chemotherapy. **b** Spindle cells with moderate-to-marked cytologic atypia and mitotic activity (high-power view). **c** Computed tomography shows the enlarged axillary lymph nodes (arrow heads). **d**, **e** T1-weighted fat-saturated contrast-enhanced magnetic resonance imaging shows a 78 × 58 mm tumor adjacent to the pectoralis major muscle with enhancement (arrow) (**d**). The center of the tumor is necrotic (*asterisk*) based on the low intensity on contrast-enhanced T1-weighted images (**d**) and high intensity on T2-weighted image (**e**). The pre-existing necrotic area is considered “pre-treatment necrosis” to differentiate it from post-treatment necrosis caused by neoadjuvant chemotherapy

A 52-year-old Japanese post-menopausal woman was referred with a left breast mass, which had rapidly increased in size. Bleeding from her chest wall started about 6 months prior to presentation. The tumor measured 10 cm in its greatest dimension. It was non-mobile, contained an oozing skin ulcer and was fixed to her chest wall (Fig. 1a). Several ipsilateral axillary lymph nodes were palpable. A skin biopsy was performed and showed a fascicular pattern of spindle cells (Fig. 1b). Immunohistochemical staining established the diagnosis of leiomyosarcoma. A computed tomography (CT) scan showed no evidence of metastases to other sites except the enlarged left axillary lymph nodes (Fig. 1c). Enhanced magnetic resonance imaging (MRI) on T1-weighted images showed a 78 × 58 mm tumor invading the pectoralis major muscle (Fig. 1d). The central portion of the tumor was necrotic, based on the low intensity signal on T1-weighted images (Fig. 1d) and high intensity signal on T2-weighted images (Fig. 1e).

Based on the results of the European sarcoma trial, we decided to administer three cycles of doxorubicin (30 mg/m^2^ on day 1, day 2) plus ifosfamide (2000 mg/m^2^ on days 1 to 5) with mesna uroprotection (400 mg/m^2^ × 3 on days 1 to 5). We explained to our patient the treatment strategy and got written informed consent. She completed this regimen without major adverse effects. After neoadjuvant chemotherapy, there was no significant change in the size and enhanced pattern on MRI, even though at the completion of neoadjuvant chemotherapy the tumor became mobile.

**Fig. 2:**
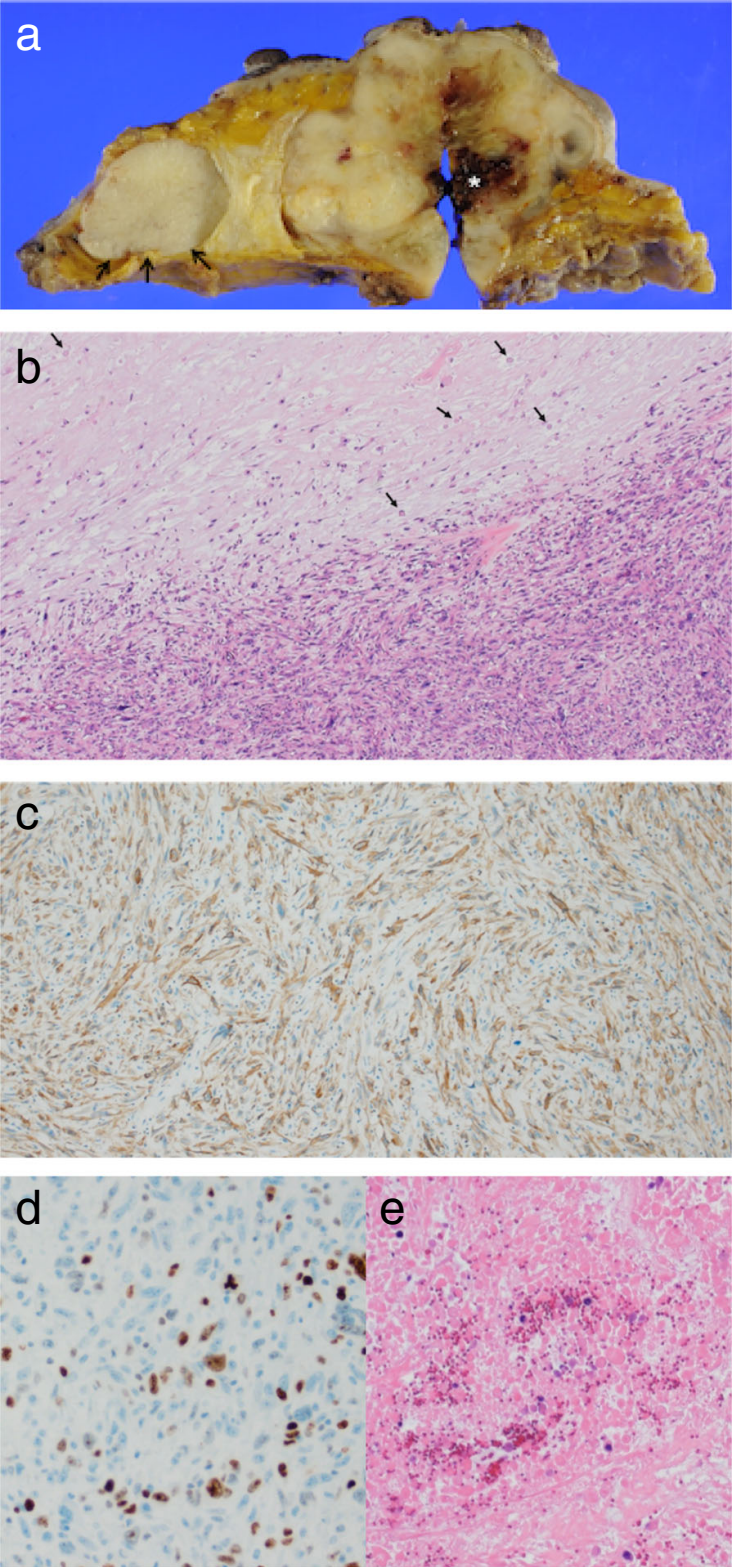
**a** A cross-sectional view of the tumor. Pre-treatment necrosis and hemorrhage is recognized in the center of tumor (*asterisk*). The mass to the medial side of the main tumor is a benign fibroadenoma (*arrow*). **b** On histological examination, nuclear atypia and tight fascicular proliferation of highly cellular spindle tumor cells in the hypercellular areas (high-power view). It also shows the necrotic fibrosis and the weakly stained nucleus known as ghost cells caused by neoadjuvant chemotherapy in hypocellular areas (*arrow*). **c** The neoplastic cells are reactive for alpha smooth muscle actin on immunohistochemistry. **d** The Ki-67 labelling index was approximately 20%. **e** Necrotic tissue is replaced by granulation and fibrous connective tissue (high-power view)

A left mastectomy with axillary lymph node dissection was performed. On gross examination, the tumor measured 9 × 7 × 6 cm. The cut surface revealed a gray-white and fleshy tumor with areas of hemorrhage and necrosis with calcification, and the tumor protruded through the skin (Fig. 2a). On histological examination, the main tumor consisted of bundles of spindle cells with well-defined bright eosinophilic cytoplasm, and pleomorphic nuclei (Fig. 2b). There were several foci of coagulative necrosis and 10% mitoses in a high-power field. There was no epithelial component similar to ordinary ductal breast cancer. Pathological assessment revealed that the axillary lymph nodes and the surgical margin were negative. On immunohistochemical examination, the neoplastic cells were positive for α-smooth muscle actin (Fig. 2c) and desmin, and negative for AE1/AE3, CAM5.2, and S100. The Ki-67 labeling index was approximately 20% (Fig. 2d). The final diagnosis was leiomyosarcoma.

The tumor had two components including viable leiomyosarcoma and necrosis. It is hard to differentiate pre-treatment and post-treatment necrosis macroscopically. By referring to pre-neoadjuvant chemotherapy MRI scans (Fig. 1d and e), we were able to differentiate the necrotic areas of the tumor. The post-treatment necrosis was a result of the neoadjuvant chemotherapy. Ghost cells with faint nuclei and fibrosis were seen in areas of post-treatment necrosis and represented the tumor response (Fig. 2b, arrow). Areas of pre-treatment necrosis contained granulation tissue, severe fibrosis, and hemorrhage (Fig. 2e).

A follow-up contrast-enhanced CT scan of her chest and abdomen showed no residue or recurrence at 12 months. She is thriving and was disease free at 1.5-year follow-up.

## Discussion

Sarcomas arise throughout the body, including the breast, and are composed of many histological subtypes. Breast sarcomas are defined as a group of mesenchymal malignant tumors similar to other soft tissue sarcomas including angiosarcomas and excluding malignant phyllodes tumor [1]. Leiomyosarcoma derives from the smooth muscle lineage, which is one of the most common soft tissue sarcomas, accounting for between 10 and 20% of all newly diagnosed soft tissue sarcomas [7]. However, leiomyosarcomas of the breast are rare, with fewer than 50 cases reported in the world literature [8].

Most trials have not shown a survival benefit conferred by adjuvant chemotherapy for the treatment of soft tissue sarcomas [7]. The first evidence for a benefit was reported from a randomized trial for high-risk soft tissue sarcomas of extremities and trunk wall including leiomyosarcoma. The results of this study suggested that neoadjuvant chemotherapy with a “conventional regimen” including standard anthracycline plus ifosfamide was superior to histology-driven tailored chemotherapy for patients. This trial showed an improved overall and relapse-free survival advantage in patients who received neoadjuvant therapy [5, 6].

In the present patient, a bulky sarcoma mass invading the pectoralis major muscle suggested the use of neoadjuvant chemotherapy (anthracycline/ifosfamide) to shrink the tumor and allow negative surgical margins after resection.

Recently, neoadjuvant chemotherapy has been used more frequently to treat patients with soft tissue sarcomas. Having been treated with neoadjuvant chemotherapy, soft tissue sarcomas such as gastrointestinal stromal tumors are likely to result in necrosis, intra-tumor hemorrhage, or myxoid degeneration, which differs from malignancies not treated that way [9]. The necrotic area is divided into two parts which include pre-treatment and post-treatment necrosis. Pre-treatment necrosis was differentiated from the treatment effect in this patient by evaluating the pre-neoadjuvant chemotherapy MRI (asterisk in Fig. 1d and e) and comparing it to imaging studies obtained after treatment.

It is necessary to have a grading system to objectively monitor the regression of soft tissue sarcomas following neoadjuvant treatment, but there is no accepted standard system to date. The European Organisation for Research and Treatment of Cancer - Soft Tissue and Bone Sarcoma Group (EORTC-STBSG) proposed that therapeutic response should be evaluated by the change on pathological findings of stainable tumor cells and necrotic area for the entire tumor following neoadjuvant chemotherapy [10]. They recommended excluding pre-treatment necrosis from evaluation and categorize as follows: A no stainable tumor cells, B single stainable tumor cell, C ≧ 1% < 10% stainable tumor cells, D ≧ 10% < 50% stainable tumor cells, and E ≧ 50% stainable tumor cells.

In the present patient, after excluding pre-treatment necrosis from evaluation, 30% of the tumor was necrotic and over 50% of the area was regarded as having stainable tumor cells. According to the proposed EORTC-STBSG tumor regression grading scheme, this patient’s therapeutic grade is “E”. The EORTC-STBSG concluded that less than 5–10% of stainable tumor cells is a good responder and correlates with favorable survival [10].

Lymphatic spread is uncommon in sarcomas. In a prospective analysis of 1722 soft tissue sarcomas, lymph node metastases were present in 2.6% of patients [11]. In the present patient, axillary lymph node metastases were suspected based on physical examination and imaging studies and led us to perform axillary dissection. Pathologic evaluation showed no lymph node metastases, however. Axillary lymph nodes should be treated individually depending on the situation for that patient.

There is no consensus regarding the use of adjuvant radiation therapy, also. Radiation therapy may reduce the risk of local recurrence but does not result in a survival advantage. In a study of extremity soft tissue sarcomas, a cohort study and a retrospective study reported that postoperative radiation therapy may improve the survival advantage in large (> 5 cm) high-grade lesions [12, 13]. Due to the size of the tumor in this patient, and the fact that it was a high-grade subtype, we recommended the use of adjuvant radiotherapy. After a thorough discussion with our patient, she decided not to undergo adjuvant radiotherapy.

Outcomes of the larger and most recent series on “breast sarcomas” are provided in Table 1. One series included phyllodes tumor in the analysis [4]; most series considered it a distinct entity from breast sarcoma in view of its epithelial component [1–3, 14]. Interpretations regarding secondary sarcoma are widely divided. Some series have excluded secondary sarcoma from the analysis on the basis of its etiology [1, 14]. However, the number of secondary sarcomas is increasing due to breast irradiation for previous breast carcinoma, as breast conservation in the surgical treatment necessitates adjuvant radiotherapy [3, 4]. A greater proportion of the radiation-induced sarcomas are angiosarcomas. The proportion of angiosarcomas reported in the literature varies from 41%, 42% to 92% [2–4]. In general, breast sarcomas have a poorer prognosis than breast cancer. Five-year overall survival ranged between 44 and 67% [1, 3, 14] and 5-year sarcoma-specific survival ranged between 56.6 and 78% [1, 2, 4]. Tumor size (> 5 cm), secondary sarcoma (radiation-induced sarcoma, chronic lymphedema), residual tumor after treatment, cellular pleomorphism, and angiosarcoma were found to be prognostic factors for survival rate. In these studies, although the mainstay of treatment should be surgical excision with negative margins, neither adjuvant chemotherapy nor radiotherapy improved survival [1–4, 14].

**Table 1:** Patients’ demographics, tumor characteristic, prognostic factor, and clinical outcomes

Authors	Year of publication	Data	Number of patients	Histologic subtype	Adjuvant therapy	Prognostic factors	Clinical outcomes
Adem *et al*. [1]	2004	Single institution study	25	Fibrosarcoma, angiosarcoma, pleomorphic sarcoma > myxofibrosarcoma > leiomyosarcoma > hemangiopericytoma, osteosarcoma, excluding radiation-induced sarcoma and phyllodes tumor	1 adjuvant chemotherapy; 4 adjuvant radiotherapy	Tumor size > 5 cm	Overall survival 66% Sarcoma-specific survival 70% at 5 years
Bousquet *et al*. [3]	2007	Multicenter study	103	Angiosarcoma (including radiation-induced sarcoma) >> malignant histiocytofibroma > fibrosarcoma > liposarcoma > leiomyosarcoma > osteogenic sarcoma, excluding phyllodes tumor	11 neoadjuvant chemotherapy; 19 adjuvant chemotherapy; 50 adjuvant radiotherapy	Residual tumor after treatment, cellular pleomorphism, angiosarcoma	Overall survival 44% at 5 years
Fields *et al*. [14]	2008	Single institution study	13	Leiomyosarcoma = malignant fibrous histiocytoma = fibrosarcoma > carcinosarcoma = angiosarcoma = epithelioid cell sarcoma = rhabdomyosarcoma, excluding radiation-induced sarcoma and phyllodes tumor	5 adjuvant chemotherapy; 9 adjuvant radiotherapy	Tumor size > 5 cm	Overall survival 67% at 5 years
Pencavel *et al*. [4]	2011	Single institution study	63	Angiosarcoma (including radiation-induced sarcoma) > phyllodes tumor > dermatofibrosarcoma protuberans > Leiomyosarcoma > fibrosarcoma = pleomorphic > malignant fibrous histiocytoma = synovial	No adjuvant chemotherapy; 24 neoadjuvant radiotherapy	Radiation-induced sarcoma	Sarcoma-specific survival 93% at 2 years, 78% at 5 years
Toesca *et al*. [2]	2012	Single institution study	203	Angiosarcoma (including radiation-induced sarcoma) >> stromal sarcoma excluding phyllodes and dermatofibrosarcoma	5 adjuvant chemotherapy; 6 adjuvant radiotherapy	Secondary sarcoma (radiation-induced sarcoma, chronic lymphedema)	Sarcoma-specific survival 56.6% at 5 years

> greater than, >> much greater than, = equals

## Conclusion

For patients with soft tissue sarcomas, an *en bloc* resection with negative margins is the only potentially curative therapy. Neoadjuvant chemotherapy for aggressive high-grade lesions is widely accepted as a therapeutic option and its benefits have been reported. We believe that breast sarcomas could be treated in the same way as other soft tissue sarcomas in terms of adjuvant chemotherapy and radiotherapy. The generalized application of this approach will be difficult to assess because of the rarity of these lesions.

## Data Availability

The datasets used during the current study are available from the corresponding author on reasonable request.

## Abbreviations

CT: Computed tomography
EORTC: European Organisation for Research and Treatment of Cancer
MRI: Magnetic resonance imaging
STBSG: Soft Tissue and Bone Sarcoma Group
